# Correlates of Physical Activity in 0- to 5-year-olds: A Systematic Umbrella Review and Consultation of International Researchers

**DOI:** 10.1007/s40279-022-01761-5

**Published:** 2022-10-11

**Authors:** Jelle Arts, Elizabeth Drotos, Amika S. Singh, Mai J. M. Chinapaw, Teatske M. Altenburg, Jessica S. Gubbels

**Affiliations:** 1 Department of Public and Occupational Health, Amsterdam Public Health, Amsterdam UMC, Vrije Universiteit Amsterdam, De Boelelaan 1117, Amsterdam, The Netherlands; 2 Department of Health Promotion, NUTRIM School of Nutrition and Translational Research in Metabolism, Maastricht University, Maastricht, The Netherlands; 3 Mulier Institute, Utrecht, The Netherlands; 4 Center for Physically Active Learning, Faculty of Education, Arts and Sports, Western Norway University of Applied Sciences, Sogndal, Norway

## Abstract

**Background:**

Many children aged 0–5 years do not meet the WHO physical activity guidelines. To develop effective, evidence-based interventions, it is necessary to understand which factors are associated with physical activity in early childhood.

**Objective:**

To summarize the current evidence on correlates of physical activity in 0- to 5-year-old children.

**Methods:**

First, a systematic umbrella review was conducted following PRISMA guidelines. PubMed, Embase, PsycINFO, and SPORTDiscus were searched up to May 2020 for systematic reviews examining the association between potential correlates and quantitatively measured physical activity in children aged 0–5.9 years. Included reviews were assessed on methodological quality, and results were categorized according to the socio-ecological model. Second, 31 international researchers of physical activity in young children participated in an expert panel to reflect on the outcomes of the umbrella review and propose directions for future research.

**Results:**

Twenty-one reviews were included that examined a total of 98 potential correlates. When synthesizing all reviews, 23 correlates were found with consistent evidence for an association with a physical activity outcome. For most other potential correlates there was inconsistent evidence across reviews for associations with physical activity in young children. Although there was little overlap between the correlates identified in the umbrella review and determinants suggested by the expert panel, both confirmed the importance of socio-cultural, policy, and physical environmental factors in general.

**Conclusion:**

Multiple correlates of young children's physical activity were identified. However, various methodological challenges (e.g., measurement instruments) and the large heterogeneity (e.g., study samples, correlates, and outcome measures) hindered formulating definitive conclusions. Moreover, none of the reviews reported on the interrelatedness between correlates, which would align with more holistic understandings of behavior. Our findings indicate the urgent need for establishing a common ground in definitions, assessment methods, and analytical methods to further the field of physical activity research in this tremendously important age group.

**Prospero Registration Number:**

CRD42020184159.

**Supplementary Information:**

The online version contains supplementary material available at 10.1007/s40279-022-01761-5.

## Key Points

**Table:** 

In general, there was inconsistent evidence for associations between hypothesized correlates and physical activity in 0- to 5-year-old children.
Both the umbrella review and expert panel highlight a number of methodological challenges (e.g., assessment of physical activity) that should be addressed in future research to optimally inform physical activity promotion in young children.

## Introduction

Promoting physical activity (PA) in early childhood is critical to support the growth and development of young children and the maintenance of long-term health [1–3]. For example, PA during the early years is positively associated with motor as well as cognitive development [3–5]. Likewise, PA is beneficial to bone health, psychosocial health, cardiometabolic health indicators, and a reduced risk of obesity in early childhood [3, 6, 7]. Moreover, studies show that PA habits develop early in childhood, which emphasizes that early childhood should be targeted as a critical period to promote healthy lifestyle behaviors [8–10].

Because of the growing recognition of the importance of PA in early life, the World Health Organization (WHO) [11], as well as several individual countries (e.g., Canada [12], South Africa [13], Australia [14], and the United Kingdom [15]), have developed PA and/or 24-h movement guidelines for this age group in the past decade. Unfortunately, only a small proportion of young children meet these PA guidelines [16–19]. For example, research showed a compliance rate of less than 20% in children aged between 3 and 6 years [16, 20]. Therefore, effective interventions are needed that aim to increase PA in young children. So far, there is very limited high-quality evidence of interventions promoting PA among young children, with the subgroup of 0- to 2-year-olds especially being neglected [21]. In addition, the limited number of interventions developed for this age group require a more tailored approach to effectively increase young children's PA [22]. To develop effective, evidence-based interventions, it is vital to have an understanding of the factors that determine PA in early childhood.

While PA research in this age group is still an emerging topic, several reviews on the correlates of PA in young children have been conducted. The focus of these reviews varies in terms of age group (e.g., infants, toddlers, preschoolers), PA intensity and type (e.g., light, moderate-to-vigorous), and/or correlate category (e.g., social, demographic). So far, there is no overview available summarizing the findings of these reviews for infants (0–1 year), toddlers (1–3 years), and preschoolers (3–5.9 years). Although studies on preschoolers were included in a review of reviews on correlates of PA in children aged 3–12 years [23], results were not presented separately for preschoolers. Additionally, several new reviews on the correlates of PA in young children have been published since 2014.

Hence, a comprehensive overview of the correlates of PA during early childhood is highly warranted. In this umbrella review, we aimed to summarize findings from all available reviews regarding the correlates of PA in children aged < 6 years old. In addition, we aimed to enrich this umbrella review with the perspectives of an expert panel consisting of international researchers on this topic. Based on both the literature and consultation of international researchers, we (1) summarize evidence on factors that potentially determine young children’s PA, (2) identify gaps in the literature, and (3) propose directions for future research.

## Methods

The current study followed a two-step procedure: (1) conduct an umbrella review on correlates of PA in 0- to 5-year-olds, and (2) consult a panel consisting of international researchers of PA in young children to reflect on the outcomes of the umbrella review and propose future directions for research.

We registered the umbrella review on PROSPERO (international prospective register of systematic reviews; registration number CRD42020184159) and followed the Preferred Reporting Items for Systematic reviews and Meta-Analyses (PRISMA) guidelines [24].

### Umbrella Review

#### Search Strategy

We searched four electronic databases on 4 May 2020: PubMed, Embase, PsycINFO, and SPORTDiscus. We applied no language restrictions, publishing date limits, or other filters during the search. Search terms related to the target population (e.g., infant, preschool, “early childhood”), physical activity (e.g., exercise, movement), determinants and/or correlates (e.g., association), and the desired article type (e.g., “systematic review”). We used MeSH terms and PsycINFO thesaurus terms where appropriate. The full search strategy is available in the Electronic Supplementary Material (ESM, Online Resource 1).

#### Study Selection

We developed the following eligibility criteria using the PICOS (Patient, Intervention, Comparison, Outcomes, and Study) framework [25]: articles were included in our umbrella reviews when they (1) were peer-reviewed, published review articles in English; (2) examining the association between one (or multiple) potential determinant(s)/correlate(s) and quantitatively measured PA; (3) in (apparently) healthy, typically developing children aged within the range 0–5.9 years or of an average age ≤ 5.9 years at follow-up. We also considered reviews that investigated a potential reverse association between PA and an outcome, if cross-sectional studies were reported on separately, since the direction of the relationship in such studies is not apparent. Reviews of interventions were included if these interventions focused solely on PA as the outcome, excluding integrated obesity prevention interventions (e.g., focusing on diet in addition to PA). Additional exclusion criteria were the consideration of solely prenatal correlates and focusing solely on children born preterm. We also excluded umbrella reviews (e.g., reviews of reviews).

We imported all records from the search into the Rayyan web application for the screening process [26]. First, we removed duplicates, and then two reviewers independently screened titles and abstracts, discussing conflicts until a consensus was reached (E.D. and either J.G. or A.S). Next, we performed a full-text screening against eligibility criteria on the remaining articles by a single reviewer (E.D.), noting reasons for exclusion. Two additional reviewers (J.G. and A.S.) each independently screened a random selection (20%) of the full-text articles to confirm choices of inclusion/exclusion. We hand-searched reference lists of included reviews for additional eligible articles.

#### Data Collection and Synthesis

We used a piloted spreadsheet to extract data from the selected studies. The data extraction form included the following data: study year and authors, review type, review aim, number of selected studies, study designs of included studies, number and characteristics of study participants, any methodological quality appraisal instruments used, correlates/determinants and their categorization, PA outcome measures, and results. We also extracted the number of studies using direct (e.g., accelerometer, heart rate monitor, doubly labeled water, direct observation) versus indirect measurement instruments (e.g., proxy-report questionnaires). Two reviewers independently performed the data extraction (E.D. and either J.A. or Maxine de Jong; research assistant), and resolved disagreements through discussion.

We carried out the methodological quality appraisal using an adapted version of the modified AMSTAR tool developed by Pollock et al. in 2014 [27]. This tool uses the 11 items of the original AMSTAR tool, and provides clearly defined, dichotomous sub-questions to clarify when a ‘yes,’ ‘no,’ ‘unclear,’ or ‘partial’ designation should be awarded [28]. We chose to further subdivide some items to gain better insight into specific issues found in the reporting of the reviews (i.e., items 2, 3, 4, 6, and 9). The final adapted quality appraisal tool is available in the ESM (Online Resource 2). Two reviewers (E.D. and J.A.) appraised the quality of included reviews independently, and any disagreements were resolved through consensus, referring to a third reviewer when necessary (either J.G. or A.S.).

We categorized variables using five adapted categories of the socio-ecological model applied by Sallis and colleagues: demographic/biological, behavioral attributes/skills, social/cultural/policy, physical environmental, and psychological/cognitive/emotional factors [29]. When possible, we extracted and reported separate results for specific outcome measures of PA, i.e., total physical activity (TPA), moderate-to-vigorous physical activity (MVPA), or light physical activity (LPA). When reviews reported results of PA outcome measures other than TPA, MVPA, or LPA (e.g., tummy time) or compiled data across multiple PA outcome measures, we listed these results in the ‘varied PA’ category.

We listed a review in our results for any possible correlate of PA when it included two or more studies that examined this potential correlate. There is some inconsistency with how the terms ‘correlate’ and ‘determinant’ have been used in the literature [30]. Since mediators, moderators, and confounders can act to influence measured changes in PA, using the term determinant might be misleading since this implies a cause-and-effect relationship [30]. Therefore, as proposed by Bauman et al. (2002), in this umbrella review the term ‘correlate’ is used, instead of determinant, to describe statistical associations between measured variables and PA [30]. We used the following summary codes for associations observed in the individual reviews:

- Evidence for a positive (+) or negative (−) association: if 60–100% of the studies within a review found a significant association in the reported direction;
- Mixed evidence (+ /−): if 33–59% of the studies within a review found a significant association in the reported direction;
- No evidence for an association (0): if 0–32% of the studies within a review found a significant association in the reported direction;
- Unclear (?): if the total number of studies examining the variable was unclear, even if the review itself concluded a positive or negative association.

In addition, we also extracted meta-analytic data including correlation coefficients, 95% confidence intervals and *p* values, if available in reviews. Strengths of correlations were categorized based on Cohen’s recommendations for effect sizes: a correlation of 0.09 or less was considered a null effect, 0.10 a small effect, 0.30 a medium effect, and 0.50 a large effect [31].

Next, when synthesizing all reviews, we considered there to be ‘consistent evidence’ for an association between a variable and a PA outcome when the association in the reported direction was found in the majority (i.e., 51%) of reviews, and there was no review reporting no evidence or evidence for an association in the opposite direction. When all reviews found no evidence for an association between a variable and PA, we considered this as ‘consistently no evidence’ for an association. When both criteria were not met, we considered this as ‘inconsistent evidence’ for an association with PA (e.g., one review found evidence for an association with PA, but the majority of the reviews did not find an association).

### Consultation of International Researchers

#### Participants

We approached international researchers in the field of PA in young children to participate in the expert panel if they: (1) had been active as a researcher for > 5 years in the field of PA in children aged 0–5 years old (years of experience self-reported by researcher); (2) published in the field of PA in children aged 0–5 years; and (3) were able to answer online surveys in English to take part in the panel.

Participants were recruited in three rounds:

- First recruitment round: The authors of this umbrella review were asked to each independently recommend three to five international researchers of PA in children aged 0–5 years old. After removing overlap, this resulted in a convenience sample of 21 individual names that were invited for participation. Researchers (invitees of the first recruitment round) were also asked to each recommend three additional researchers with expertise in PA of children aged 0–5 years (i.e., snowball sampling).
- This procedure was repeated for the invitees of the second and third recruitment round.

This resulted in a total of 41 invited researchers in the first round of the expert panel.

We sent out the link to the first online survey in the last week of March 2021. We asked invited researchers to answer the survey within one week. After one week, we sent a reminder. The first-round survey was accessible for 10 working days, resulting in 31 respondents in the first round. The link to the second round was sent out in the last week of May 2021. Similar to the first round, we asked researchers to answer the survey within a week and sent out a reminder after 1 week. The survey was accessible for 10 working days, resulting in 21 respondents.

#### Procedure for the Expert Panel

The expert panel consisted of two rounds. In the first round we presented the list of potential correlates that we identified from the systematic umbrella review in the five categories of the socio-ecological model (i.e., demographic/biological, behavioral attributes/skills, social/cultural/policy, physical environmental, and psychological/cognitive/emotional). We asked participating researchers to indicate whether the list was complete, and if not, to list the missing determinants they considered relevant. Note that throughout the expert panel, we asked participating researchers to share their knowledge and reflections with regard to determinants instead of correlates, as we were specifically interested in their perspective on variables directly influencing PA. Finally, we asked participating researchers to select a maximum of ten determinants from the combined list of variables (i.e., potential correlates identified in the umbrella review and their own suggestions for determinants) that they considered most important for influencing young children’s PA.

In the second round we presented (1) a summary of the determinants considered important by researchers in the first round and (2) the results from the umbrella review (i.e., correlates that were consistently associated with PA). Subsequently, we asked researchers which discrepancies between the findings from the expert panel and the umbrella review they considered to be the most notable. Additionally, we asked which of the following topics need to be addressed in future research into early childhood PA: (1) the definition of PA behavior in young children, (2) measurement instruments, (3) research designs, (4) un(der)studied determinants, (5) data analyses, or (6) other topics (open answer). We concluded by asking them to prioritize these future directions.

## Results

### Umbrella Review

**Fig. 1:**
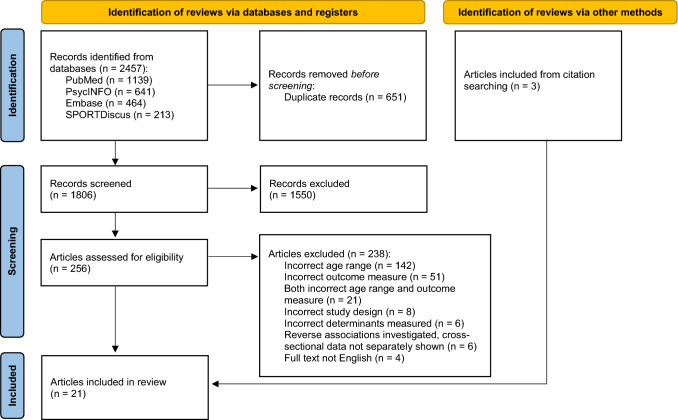
Preferred Reporting Items for Systematic reviews and Meta-Analyses (PRISMA) flow diagram of study inclusion

The literature search retrieved 2457 articles. Following the screening and selection process, we included 18 reviews from the search, as well as three additional reviews identified through citation searching, resulting in a total of 21 reviews (Fig. 1). Three of the selected systematic reviews also included meta-analyses [32–34]. Table 1 summarizes the characteristics of the individual reviews.

**Table 1:** Characteristics of included reviews

Study	Objective	Publication type (# of relevant studies/total studies) Study publication dates: Range of years	Study designs included (#)	Participant range (mean or median if available) Characteristics	Study sample size range of included studies Total participants (if compiled and reported)	Correlate categories examined (correlate category that was a focus of the review)	PA outcome measure(s) Direct vs. indirect measurement instrument (#) ^a^
Bingham et al. 2016 [39]	To synthesize studies examining potential correlates and determinants of PA during the early years of life, accounting for different forms of PA measurement	Systematic Review (130/130) Years NR	Cross-sectional (118) Longitudinal (12)	0.5–5.95 y (mean = 4.3 y) Not yet in formal primary school	20–10,694	Demographic/biological Behavioral attributes/skills Social/cultural/policy Physical environmental	Total PA, MVPA, LPA Direct (104) Indirect (24) Unclear (2)
Broekhuizen et al. 2014 [46]	To provide overview of evidence regarding the value of (pre)school playgrounds for children's health, including PA (and cognitive and social outcomes); identify most effective playground characteristics	Systematic Review (9/30) 2000–2012	Experimental (4) Observational (5)	2–18 y (reports separately on 2–6 y) Preschool students	5–783	Social/cultural/policy Physical environmental *(Preschool playground factors)*	Varied PA Measures Direct (9)
Carson et al. 2017 [49]	To examine the associations between objectively and subjectively measured PA and health indicators in the early years	Systematic Review (55/96) 1984–2016	Cross-sectional (55) [Examined reverse association]	0–4.99 y	100–8,834	Demographic/biological Behavioral attributes/skills Psychological/cognitive/emotional	TPA, MPVA, LPA, varied PA Direct (22) Indirect (28) Both (5)
Chaput et al. 2017 [52]	To examine the relationships between sleep duration and various health indicators [including PA] in children aged 0–4 years	Systematic Review (3/69) 2012–2016	Cross-sectional (3) [Examined reverse association]	0–4 y	240–1,028 Total 2,272	Behavioral attributes/skills *(Sleep-related factors)*	TPA (and NR) Direct (2) Indirect (1)
Costa et al. 2019 [45]	To synthesize research on the longitudinal relationship between non-parental childcare in the early years and PA (and diet, sleep, and sedentary behavior)	Systematic Review (2/13) 2009–2013	Longitudinal (2)	3–6 y Not yet in formal primary school	658–6,550	Physical environmental (*Non-parental childcare)*	Varied PA Direct (1) Indirect (1)
De Craemer et al. 2012 [35]	To review the correlates of PA, sedentary behavior, and eating behavior in children between 4–6 years old	Systematic Review (20/43) Years NR	NR (separately for PA studies) [Included both longitudinal and cross-sectional studies]	4–6 y	NR	Demographic/biological Behavioral attributes/skills Social/cultural/policy Physical environmental	Total PA, MVPA, PA during recess Direct (15) Unclear (5)
Hesketh et al. 2017 [36]	To synthesize the quantitative literature from prospective and interventional studies to ascertain the determinants of change in PA in younger children; to establish which determinants are associated with change and at which levels of influence these factors operate	Systematic Review (42/42) 2004–2014	Longitudinal (4) Experimental (38)	3–6 y (at baseline) Follow up 1–5 y later	72–244	Demographic/biological Behavioral attributes/skills Social/cultural/policy Physical environmental Psychological/cognitive/emotional	Varied PA Direct (32) Indirect (10)
Hoyos- Quintero & Garcia- Perdomo, 2019 [40]	To examine the correlates/determinants of factors (biological, demographic, sociocultural, and environmental) on PA levels in early childhood	Systematic Review (14/14) 2003–2018	Experimental (2) Longitudinal (4) cross-sectional (8)	2–6 y	32–2026 Total 6,704	Demographic/biological Behavioral attributes/skills Social/cultural/policy Physical environmental Psychological/cognitive/emotional	Varied PA Direct (9) Indirect (2) Both (3)
Hewitt et al. 2017 [37]	To examine the correlates of tummy time of objectively and subjectively measured tummy time in infants (aged 0–12 months) across observational study designs	Systematic Review (16/16) 1965–2016	Experimental (2) Longitudinal (8) Cross-sectional (6)	0–12 mo	20–542 Total 2,372	Demographic/biological Behavioral attributes/skills Social/cultural/policy Physical environmental	Tummy time Direct (7) Indirect (9)
Hinkley et al,. 2008 [42]	To investigate comprehensively the correlates of preschool children’s PA, through social–ecological models	Systematic Review (24/24) 1980–2006	Longitudinal (3) Cross-sectional (21)	2–5 y Not in formal schooling yet	30–3,141	Demographic/biological Behavioral attributes and skills Social/cultural/policy Physical environmental Psychological/cognitive/emotional	Varied PA Direct (10) Indirect (10) Both (4)
Janssen et al. 2020 [38]	To determine how screen time, sedentary time and PA are associated with eight sleep outcomes (i.e., total sleep duration; night awakenings; sleep onset latency; bed time; daytime napping; sleep efficiency; sleep stability; and sleep quality) in children aged 0–4 years	Systematic Review and Meta-Analysis (9/31) 2011–2019	Cross-sectional (9) [Examined reverse association]	0–5 y	48–1,028	Behavioral attributes/skills *(Sleep-related factors)*	TPA, MVPA, varied PA Direct (5) Indirect (3) Both (1)
Li et al. 2015 [43]	To conduct a systematic review of longitudinal studies examining the factors related to PA in young children	Systematic Review (9/9) 1980–2013	Longitudinal (9)	2–6 y	17–628	Demographic/biological Social/cultural/policy Physical environmental	Varied PA Direct (9)
Logan et al. 2015 [51]	To synthesize the evidence of the relationship between fundamental motor skills competence and PA	Systematic Review (4/13) 2008–2013	Cross-sectional (4)	3–5 y	34–198	Behavioral attributes/skills *(Motor skills)*	Varied PA Direct (4)
Marshall et al. 2004 [34]	To review the empirical evidence of associations between television (TV) viewing, video/computer game use and (a) body fatness, and (b) PA	Meta-Analysis (3/33) Years NR	NR (study type not stratified by age category)	0–6 y	NR Total 631	Behavioral attributes/skills *(TV viewing)*	NR
Pearson et al. 2014 [32]	To systematically review and meta-analyse peer-reviewed research describing the association between sedentary behavior and PA in children and adolescents	Meta-Analysis (12/163) 1993–2012	Longitudinal (2) cross-sectional (10)	0–5 y	NR	Behavioral attributes/skills *(Sedentary behavior)*	Varied PA Direct (5) Indirect (7)
Tonge et al. 2016 [50]	To identify correlates of PA in early childhood education and care services	Systematic Review (27/27) 1992–2014	Quantitative studies (27) [no further details]	0–6 y	31–1,352	Demographic/biological Behavioral attributes/skills Social/cultural/policy Physical environmental	Varied PA Direct (27)
Tucker, 2008 [41]	To present research on the PA levels of preschool-aged children (aged 2–6 years)	Systematic Review (39/39) 1986–2007	Quantitative studies (39) [No further details]	0–6 y Preschoolers	12–3,141 Total 10,316	Demographic/biological Physical environmental	Varied PA Direct (29) Indirect (6) both (4)
Ward et al. 2015 [47]	To identify if childcare educators’ practices predict or are associated with preschoolers’ PA and eating behaviors in childcare centers and to assess the effectiveness of interventions that control educators’ practices or behaviors in order to improve preschoolers’ PA and eating behaviors	Systematic Review (10/15)	Cross-sectional (4) Experimental (6)	NR (age) In preschool	5–885	Social/cultural/policy Physical environmental *(Childcare educator practices)*	Varied PA Direct (9) Both (1)
Ward et al. 2016 [48]	To systematically review how preschoolers’ eating behaviors and PA relate to their peers’ behaviors, and discuss avenues for future research	Systematic Review (6/13) 1972–2014	Experimental (3) Cross-sectional (3)	2–6 y In preschool	20–892	Social/cultural/policy *(Peer behavior)*	Varied PA Direct (6)
Xu et al. 2015 [44]	To update the current literature investigating parental influences and their associations with both PA and screen time in young children	Systematic Review (18/30) 2001–2013	Longitudinal (5) Cross-sectional (13)	0–6 y	69–10,694	Social/cultural/policy *(Parent factors)*	Varied PA Direct (8) Indirect (10)
Yao & Rhodes, 2015 [33]	To provide a cohesive and comprehensive examination of the parental correlates, and potential moderators, of child PA, and to provide a comprehensive meta-analysis that overcomes the limitations of prior narrative reviews and quantitative reviews with small samples	Systematic Review and Meta-Analysis (14/112) 1983–2014	Cross-sectional (14)	2–5.4 y	14–2,026	Social/cultural/policy *(Parent factors)*	Varied PA Direct (number not reported, but majority)

*LPA* light physical activity, *mo* months, *MVPA* moderate-to-vigorous physical activity, *NR* not reported, *PA* physical activity, *TPA* total physical activity, *y* years

^a^Direct measurement instruments include accelerometry, pedometry, direct observation, doubly labeled water, heart rate monitoring; Indirect measurement instruments include parent/teacher proxy-report, questionnaires

#### Characteristic of Included Reviews

Fourteen reviews were published in the past 5 years. The earliest publication we included dated from 2004: a meta-analysis focusing on the relationship between TV watching/video game use and PA [34]. The included reviews examined studies from 1972 to 2019, though three did not explicitly list the publication dates of included studies [32, 34, 35]. The individual studies were mostly conducted in North America, Europe, Australia, and New Zealand. Studies from Asia were less frequent (reported in six reviews [33, 35–38]), and four reviews contained a few studies from Africa and/or South America [37, 39–41].

The majority of the data presented in the reviews were from cross-sectional studies, though nine reviews also included longitudinal studies [32, 35–37, 39, 40, 42–45] and six reviews also included intervention/experimental studies [36, 37, 40, 46–48]. One review included solely longitudinal studies [45], and three reported data on longitudinal studies separately [36, 39, 43]. The number of studies included in each review ranged from 2 [45] to 130 [39]. Nine reviews had age ranges that included infants (0–1 year), toddlers (1–3 years), and preschoolers (3–5.9 years) [19, 32, 34, 38, 39, 41, 44, 49, 50], six included both toddlers and preschoolers [33, 40, 42, 43, 46, 48], four contained samples of only preschoolers [35, 36, 45, 51], one focused exclusively on infants [37], and in one review the age of the participating children was not clearly reported, though all participants were in preschool [47].

Nine reviews examined a wide range of correlates. The remaining reviews focused on specific correlates, for example regarding a specific behavior (e.g., sleep-related behaviors [52], TV viewing [34]) or a specific setting (e.g., playground factors [46]). The majority of the reviews compiled the results of various PA outcome measures as a single outcome, while others reported data for specific PA outcome measures, such as TPA, MVPA, or LPA. Most reviews included a combination of studies with either direct or indirect PA measurement instruments and/or studies that combined both. Five reviews exclusively included studies with direct PA measurement instruments [43, 46, 48, 50, 51].

#### Quality Assessment

Table 2 presents the results of the methodological quality assessment. All reviews consistently provided ‘a priori' research designs, and 20 out of 21 reported comprehensive literature searches, though four reviews did not include information on the search period [36, 37, 42, 43]. Seventeen reviews clearly outlined eligibility criteria, but 18 reviews did not report whether or not filters were used in the literature search. Several reviews reported fully independent, duplicated study screening [32, 35, 37, 39, 40, 46–47, 49, 50, 52] and data extraction [32, 36, 40, 46, 47], though some only duplicated a portion of the screening [33, 36, 43, 48] and/or data extraction [34, 37, 38, 45, 48, 49, 52]. Reviews often listed correlates but did not provide further information on the specific outcomes and/or measurement instruments used for these correlates. For the PA outcomes, all except for one review [34] provided information on both PA outcomes and measurement instruments. Six of the 21 reviews did not perform assessments of the methodological quality of included studies [34, 35, 41, 42, 50, 51]. Of the remaining 15 reviews, nine completed fully independent quality assessments by two researchers [32, 38–40, 43–47]. Reviews mentioned study quality in the formulation of conclusions for all reviews that performed a quality assessment, though sometimes only briefly (e.g., by recommending more high-quality research). None of the reviews weighed the methodological quality or differentiated between studies of different quality when formulating conclusions. Nine reviews did not acknowledge or assess heterogeneity, and if they did, it was often briefly in reference to not performing a meta-analysis. All three meta-analyses stated statistical definitions of heterogeneity and applied random-effects models [32–34]. Six reviews considered or acknowledged publication bias, with two performing a statistical test for publication bias [32, 33].

**Table 2:** Methodological quality assessment of included reviews

Adapted AMSTAR item	[39]	[46]	[49]	[52]	[45]	[35]	[36]	[40]	[37]	[42]	[38]	[43]	[34]	[32]	[50]	[41]	[47]	[48]	[44]	[33]	[51]
1. 'A priori' design	Y	Y	Y	Y	Y	Y	Y	Y	Y	Y	Y	Y	Y	Y	Y	Y	Y	Y	Y	Y	Y
2a. Duplicate study selection	Y	Y	Y	Y	Y	Y	P	Y	Y	U	U	P	U	Y	Y	N	Y	P	U	P	U
2b. Duplicate data extraction	U	Y	P	P	P	U	Y	Y	P	U	P	U	P	Y	U	N	Y	P	U	U	U
3a. Comprehensive literature search (databases and other methods)	Y	Y	Y	Y	Y	N	Y	Y	Y	Y	Y	Y	Y	Y	Y	Y	Y	Y	Y	Y	Y
3b. Comprehensive literature search (years searched)	Y	Y	Y	Y	Y	Y	N	Y	N	N	Y	N	Y	Y	Y	Y	Y	Y	Y	Y	Y
4a. Inclusion criterion (no use of filters)	U	U	N	N	Y	U	U	Y	N	U	Y	U	U	U	U	U	U	N	N	U	U
4b. Inclusion criterion (language and publication type)	Y	Y	Y	Y	Y	Y	N	N	Y	Y	Y	Y	Y	N	Y	Y	Y	Y	Y	N	Y
5. List of included/excluded studies	Y	N	Y	Y	Y	N	Y	Y	Y	N	N	Y	N	N	Y	Y	Y	Y	N	Y	Y
6a. Characteristics of studies (participants)	Y	Y	N	Y	Y	N	Y	N	Y	Y	N	Y	N	Y	N	Y	N	Y	N	Y	Y
6b. Characteristics of studies (exposures/factors)	Y	Y	Y	Y	Y	N	Y	N	Y	N	N	Y	N	Y	N	Y	N	Y	Y	Y	Y
6c. Characteristics of studies (outcomes)	Y	Y	Y	Y	Y	Y	Y	Y	Y	Y	Y	Y	N	Y	Y	Y	Y	Y	Y	Y	Y
7. Quality assessed and documented, tool used	Y^1^	Y^2^	P^3^	P^4^	Y^5^	N	U^6^	Y^7^	P^8^	N	Y^3^	Y^9^	N	Y^10^	N	N	Y^11^	U^11^	Y^12^	U^13^	N
8. Quality considered in conclusions	Y	Y	Y	Y	Y	na	Y	Y	Y	na	Y	N	Na	Y	na	na	Y	Y	Y	Y	na
9a. Appropriate methods to combine studies	b	b	f	e	a	b	b	a	b	b	c	a	D	d	b	a	a	A	f	d	a
9b. Test heterogeneity and/or appropriate meta-analysis	N	N	Y	Y	Y	N	Y	U	N	N	Y	N	Y	Y	Y	N	Y	Y	Y	Y	N
10. Consideration of publication bias	N	N	N	Y	N	N	Y	N	N	N	Y	N	N	Y*	N	N	N	N	N	Y*	N
11. Conflicts of interest stated	Y	Y	Y	Y	Y	Y	Y	N	Y	Y	Y	N	N	Y	Y	N	Y	Y	Y	Y	N

*N* no,* P* partial,* U* unclear*, Y* yes, * with statistical test

a: Narrative, b: Correlate table with summary codes, c: Correlate table with summary codes and p-values by study type, d: Meta- analysis, e: Compiled by study type and outcome, f: List of supporting studies

^1^adapted CONSORT [77]/STROBE [78], ^2^adapted scales of Prins et al. [79]/De Vries et al. [80], ^3^GRADE [81], ^4^Cochrane [82]/GRADE [83], ^5^NEL-BAT [84], ^6^adapted from EPPI/Wijndaele et al. [36], ^7^Cochrane/MINORS [85], ^8^Cochrane [82], ^9^adapted scale of Uijtdewilligen et al. [86], ^10^author-developed scale [32], ^11^EPHPP Quality Assessment Tool for Quantitative Studies [87], ^12^adapted STROBE [78], ^13^adapted tool from Downs and Black[88]

#### Evidence Synthesis

In total, 98 variables were identified as potential correlates across all categories of the social-ecological model [29]. Table 3 provides a summary of the variables included in our umbrella review and their association with PA, sorted by category of the social-ecological model. Table 4 presents the results from meta-analyses.

**Table 3:** Summary of potential correlates of physical activity (PA) in 0- to 5-year-old children, sorted by category of the social-ecological model

Variable	PA outcome			
	TPA	MVPA	LPA	Varied PA
**Demographic/biological**				
Age	0: De Craemer (13) P ± : Bingham (39), **Bingham (4)**	0: De Craemer (11) P ± : Bingham (21)		+ : Hewitt (9) I, Tonge (11) 0: Hinkley (8) T/P ± : **Li (8) T/P** ?: Hoyos-Quintero & Garcia- Perdomo (NR) P
Sex (male)	+ : **Bingham (3)** 0: De Craemer (14) P ± : Bingham (77), **Hesketh (2) T/P**	+ : Bingham (54), De Craemer (6) P ± : **Bingham (2), Hesketh (2) T/P** ?: Hoyos-Quintero & Garcia-Perdomo (NR) P	± : Bingham (14)	+ : Hinkley (15) T/P, Tonge (18), Tucker (18) ± : **Li (8) T/P**, Hewitt (2) I
Ethnicity (White)	0: **Bingham (2)** ± : Bingham (18), De Craemer (2) P	0: Bingham (7) ± : De Craemer (3) P		0: Hinkley (6) T/P ± : Tonge (7)
SES	0: Bingham (7), De Craemer (9) P	± : De Craemer (9) P		0: Hinkley (3) T/P
Parental education	0: Bingham (18)	0: Bingham (13)	0: Bingham (5)	± : Tonge (3)
Parental age	0: Bingham (7), De Craemer (2) P			
Family structure	0: Bingham (8)			
Siblings (no. or order)	0: Bingham (8)			
Adiposity/BMI	0: Bingham (35), **Bingham (3)** ± : Carson (20)	0: Bingham (38) ± : Carson (17)	0: Bingham (7), Carson (8)	0: Hinkley (7) T/P, **Li (2) T/P** ± : Tonge (6)
Parental BMI	0: Bingham (12) ± : De Craemer (3) P	0: Bingham (7)		± : Hinkley (6) T/P
Fitness	+ : Carson (2)			
Physical health	± : Bingham (7)	± : Bingham (9)		
Birth weight	0: De Craemer (2) P			
Bone/skeletal health	+ : Carson (3)	+ : Carson (3)		
Cardiometabolic health (blood pressure and cholesterol)	0: Carson (4)			
**Behavioral attributes and skills**				
Motor skills	0: **Hesketh (3) T/P** ± : Bingham (23), Carson (6)	+ : **Hesketh (4) T/P**, Carson (4), De Craemer (2) P, Logan (3) P ± : Bingham (26)	0: Carson (3)	+ : Tonge (4)**,** Carson (3) I, **Hesketh (3) T/P**, Logan (4) P ± : Hinkley (3) ?: Hoyos-Quintero & Garcia- Perdomo (NR) P
TV viewing and/or sedentary behavior	± : Bingham (16), De Craemer (6) P	0: Bingham (4)		0: Marshall (3) ^c^, Pearson (19) ^c^ ± : Hinkley (7) T/P
Participation in organized sports	± : De Craemer (2) P			0: Hinkley (2) T/P
Monitoring (intrapersonal)				0: **Hesketh (3) T/P**
Sleep duration	± : Janssen (2) T	± : Janssen (2) P		+ : Chaput (3) ± : Janssen (2) P
Sleep quality	+ : Janssen (2) T	± : Janssen (2) T		
Prone sleeping				+ : Hewitt (6) I
Mean daily exposure to prone position/tummy time	0: **Bingham (2)**			± : Hewitt (4) I
Parents’ PA/PA role-modelling	± : De Craemer (6) P	+ : **Hesketh (2) T/P—Maternal PA only** 0: **Hesketh (2) T/P**		+ : Hinkley (6) T/P, Xu (10), **Li (4) T/P,** Yao & Rhodes (9) ^a^ ?: Hoyos-Quintero & Garcia-Perdomo (NR) P
Parents’ PA/family interactions	± : Bingham (17) 0: **Bingham (6)**	± : Bingham (8)		
Parental support	± : Bingham (14)			+ : Xu (11), Yao & Rhodes (7) ^b^
Parental work status	0: Bingham (15)	± : Bingham (6)		
Parenting practices	0: Bingham (19)			
Parent perceptions/beliefs about PA	± : Bingham (9)			± : Xu (2)
Parents’ barriers	± : Bingham (7)			
Parents’ PA optimism	± : **Bingham (3)**			
Parents’ PA self-efficacy	0: **Bingham (2)**			**0: Hesketh (4) T/P**
Parental PA future expectations	0: **Bingham (2)**			
Parental TV use	0: **Bingham (2)**			
Parental TV self-efficacy	0: **Bingham (2)**			
Parental screen time	0: **Bingham (2)**			
Parent encouragements	0: De Craemer (4) P			+ : Yao & Rhodes (5) ^a^ ± : Hinkley (6) T/P
Parent discouragements				0: Hinkley (3) T/P
(Perceived) PA competence	± : De Craemer (4) P	+ : De Craemer (2) P		+ : Xu (2)
Parental monitoring				+ : **Hesketh (6) T/P**
Parent motivation	± : **Hesketh (2) T/P**			0: **Hesketh (2) T/P**
Parent goal-setting				± : **Hesketh (4) T/P**
Parent knowledge	0: **Hesketh (7) T/P**	0: **Hesketh (5) T/P**		± : **Hesketh (10) T/P**
Parent skills	± : **Hesketh (2) T/P**	0: **Hesketh (3) T/P**		± : **Hesketh (2) T/P**
Parent social support	0: **Hesketh (2) T/P**			+ : **Hesketh (3) T/P**
TV viewing rules		± : De Craemer (2) P		+ : Xu (3)
Play rules	−: De Craemer (2) P			−: Hinkley (2) T/P
Time playing outside with adults	0: **Bingham (2)**			
Time spent playing with parent	+ : **Bingham (4)**			+ : **Li (3)**
Time spent playing with peers	0: **Bingham (5)**			+ : Ward-2016 (6) T/P ± : Tonge (4)
Time spent with older children	0: **Bingham (2)**			
Peer prompts				± : Tonge (2)
Opportunities for play				+ : **Hesketh (2) T/P**, Tonge (5)
Sedentary opportunities at ECE				0: Tonge (3)
ECE teacher education/training	0: **Hesketh (5) T/P**	+ : **Hesketh (9) T/P**		± : Hinkley (2) T/P, **Hesketh (2) T/P**, Tonge (8)
ECE provider knowledge				0: **Hesketh (2) T/P**
ECE teacher PA promoting practices				+ : Ward-2015 (4)
ECE educator confidence and enjoyment				0: Tonge (2)
ECE educator behavior (prompts/feedback)				± : Tonge (7)
Additional providers at ECE				+ : **Hesketh (3) T/P**
Increases in recess duration/active time at ECE	0: **Hesketh (3) T/P**	± : **Hesketh (4) T/P**		± : **Hesketh (4) T/P**
Community awareness				0: **Hesketh (3) T/P**
ECE curriculum materials	0: **Hesketh (5) T/P**	± : **Hesketh (2) T/P**		0: **Hesketh (4) T/P**
ECE PA policy				0: Tonge (3)
ECEC service quality				± : Tonge (6)
ECE group size				± : Tonge (7)
Time outdoors/in play spaces	+ : Bingham (8) 0: **Bingham (2)** ± : De Craemer (2) P	± : Bingham (6)		+ : Hinkley (4) T/P 0: Tonge (3) ?: Hoyos-Quintero & Garcia-Perdomo (NR) P
Attending childcare	0: Bingham (4)	± : Bingham (5)		± : **Costa (2) P**
Season (summer)	± : Bingham (5)	± : Bingham (8)		
Time of the week/weekday vs. weekend	± : Bingham (15)	± : Bingham (6)		0: Hinkley (2) T/P, **Hesketh (2) T/P**
Time of day	± : Bingham (4)			± : Hinkley (2) T/P
Weather (warmer/dryer)				+ : **Li (3) T/P** ± : Hinkley (6) T/P, Tonge (2)
Month	0: Bingham (6)			
Individual preschool	+ : Bingham (6)	+ : Bingham (4) ± : De Craemer (2) P		+ : Hinkley (4) T/P
Play equipment at home	0: **Bingham (2)**			
TV in home	0: **Bingham (2)**			
Convenience of play spaces				+ : Hinkley (2) T/P
Equipment (unspecified)	0: De Craemer (4) P	0: De Craemer (2) P		
Outdoor balls and play objects	+ : De Craemer (2) P			± : Broekhuizen (2) T/P
Portable equipment				0: **Hesketh (5) T/P** ± : Ward 2015 (4), Broekhuizen (2) T/P, Tonge (13)
Teacher (recess) supervision				± : De Craemer (2) P, Tonge (6)
Playground markings				± : **Hesketh (2) T/P**
Sedentary items				0: Tonge (2)
Indoor environments				0: Tonge (3)
Outdoor environments				+ : Tonge (7)
Size of play area/playground				+ : Tonge (6), Broekhuizen (2) T/P
Natural features/surfaces				± : Tonge (5)
Gradient				± : Tonge (2)
Fixed equipment				± : Tonge (10), Broekhuizen (2) T/P
Preschool location				0: Tonge (6) T/P
Preschool type				± : Tonge (5)
Field trips				± : Tonge (3)
Electronic media				± : Tonge (3)
Playground surfaces with green vegetation				± : Broekhuizen (2) T/P
Riding toys				0: Broekhuizen (2) T/P
**Psychological/cognitive/emotional**				
Knowledge (of child)	**0: Hesketh (4) T/P**	**0: Hesketh (3) T/P**		
Psychosocial health	± : Carson (2)	± : Carson (2)		
Cognitive development	± : Carson (2)			

*ECE* early childhood education, *ECEC* early childhood education and care, *LPA* light physical activity, *MVPA* moderate-to-vigorous physical activity, *PA* physical activity, *TPA* total physical activity

I—infant (0–1 year), and indicates tummy time, T—Toddlers (1–3 years), P—Preschoolers (3–5.9 years)

(total number of studies measuring an association within a review)

Bold text indicates data from longitudinal studies

+ : evidence for a positive association, −: evidence for a negative association ± inconsistent evidence, 0: no evidence for an association, ?: if the total number of studies examining the variable was unclear

^a^Meta-analysis showing small effect (*r* = 0.1–0.29)[31]

^b^Meta-analysis showing medium effect (*r* = 0.3–0.49)[31]

^c^Meta-analysis showing null effect (*r* =  < 0.1) [31]

**Table 4:** Meta-analytic results of associations of potential correlates with varied physical activity (PA) outcome measures

Study	Age range	Variable *(socio-ecological model category)*	Number of samples	Effect size (95% confidence interval)	Direction/strength of association^a^
Marshall et al. (2004) [34]	0–6 years	TV viewing *(behavioral attributes and skills)*	3	*r*_c_ = − .063 (− .206–.081) *p* < .01	0
Pearson et al. (2014) [32]	0–5 years	Sedentary behavior *(behavioral attributes and skills)*	19	*r* = − .053 (− .104–.001) *p* < .05	0
Yao & Rhodes (2015) [33]	2–5.4 years	Parent modeling/parental PA *(sociocultural/policy)*	9	*r* = .25 (.06–.42) *p* < .001	+ Small effect
		Overall parental support *(sociocultural/policy)*	7	*r* = .30 (.18–.41) *p* < .001	+ Medium effect
		Parental encouragement *(sociocultural/policy)*	5	*r* = .29 (.10–.45) *p* < .001	+ Small effect

*r*_*c*_ fully corrected sample-weighted effect size

^a^We determined strengths of associations using Cohen’s recommendations for correlational effect sizes (small effect: 0.1–0.29, medium effect: 0.3–0.49, large effect: > 0.49) [31]

##### Demographic and Biological Variables

We identified 15 demographic and/or biological variables examined in multiple studies of at least one review. Sex and age were the most commonly examined. There was consistent evidence that boys tend to be more active than girls. Specifically, three of five reviews found evidence for a positive association between male sex and varied PA outcome measures [41, 42, 50], and two of three reviews with MVPA [35, 39]. Inconsistent evidence was found for an association with TPA [35, 36, 39], of which one out of two reviews examined only longitudinal studies concluding that boys tend to have higher TPA levels than girls [39]. There was inconsistent evidence for age as a correlate of PA. Two reviews [37, 50] found evidence for a positive association between age and varied PA, including one review that exclusively included studies on infants [37], but the two other reviews found mixed evidence [43] or no evidence for an association [42] across wider age groups. One review found evidence for positive associations between skeletal health and both TPA and MVPA, and between fitness and TPA [49].

For the other demographic and biological variables, when synthesizing all reviews either consistently no evidence for an association (i.e., parental age, family structure, siblings, birth weight, and cardiometabolic health) or inconsistent evidence (i.e., child body mass index (BMI)/adiposity, ethnicity, physical health, socioeconomic status, parental education, parental BMI, parental age) was found across all PA outcome measures.

##### Behavioral Attributes and Skills

We categorized eight variables as behavioral attributes or skills. Seven reviews studied motor skills, the most frequently assessed variable in this category [35, 36, 39, 42, 49–51]. Consistent evidence was found for a positive association between motor skills and varied PA [36, 50–51], including one review of longitudinal studies [36]. Four out of five reviews also concluded that better motor skills were associated with increased MVPA levels [35, 36, 49, 51]. Reviews reported either no evidence [36] or mixed evidence [39, 49] for an association with TPA and no evidence for an association with LPA [49]. One review identified prone sleeping as a correlate of tummy time in infants, but reported mixed evidence for an association between exposure to prone position and tummy time [37]. In addition, one review included two cross-sectional studies that concluded that toddlers with higher TPA levels had better sleep quality [38]. Inconsistent evidence or consistently no evidence for an association was found between PA and the other behavioral attributes and skills when compiled across reviews (i.e., participation in organized sports, intrapersonal (child) monitoring of PA behavior, and sleep duration).

##### Social, Cultural, and Policy-Related Variables

Forty-three variables were considered social, cultural, or policy-related. These included a wide range of variables related to parents and Early Childhood Education (ECE) providers, of which most were very specific, and only included in a single review. When synthesizing all reviews, consistent evidence for an association with parental PA was found, with four reviews finding evidence for a positive association between parental PA and varied child PA outcome measures [33, 42–44], including a meta-analysis showing a small effect [33] (Table 4). One review found evidence for an association between maternal PA and child MVPA, but not parental PA (both mother and father) and child MVPA [36]. Two other reviews reported no or mixed evidence for an association between parental PA and child TPA [35, 39].

Reviews found evidence for a positive association between parents’ perceived PA competence and both child MVPA [35] and varied child PA outcome measures [44]. There was consistent evidence that TV viewing rules are positively associated with varied PA, and play rules are negatively associated with MVPA and varied PA [35, 44]. Longitudinal data showed evidence that parental monitoring of PA [36], parental social support [36], and time spent playing with parents [39, 43] are positively associated with children’s PA levels. Most other parent-related variables showed consistently no evidence for an association with PA or inconsistent evidence when compiled across reviews. This is in contrast to the results of the meta-analytic data, which showed a positive association between most parental variables and varied child PA outcome measures, with small effect sizes [33].

Two reviews identified opportunities for play as a correlate of varied PA, of which one review included two longitudinal studies [36, 50]. Hesketh et al. found evidence for positive associations between the presence of additional ECE providers and varied PA, and between ECE teacher education and child MVPA [36]. Ward et al. found evidence for the use of PA promoting practices by the educator as a correlate for varied child PA outcome measures [47].

All other ECE-related variables reported showed consistently no evidence for an association with PA (i.e., provider knowledge, educator confidence and enjoyment, and PA policy) or inconsistent evidence (i.e., teacher education/training, educator behavior, increases in recess duration/active time, curriculum materials, service quality, and group size) when synthesizing all reviews.

##### Physical Environmental Variables

We categorized 29 variables as physical environmental, though for many only a small number of studies reported on each particular variable. Three reviews examined the individual preschool the child attended, of which one review identified evidence for an association with both MVPA and TPA [39] and one with varied PA [42]. One review reported mixed evidence between individual preschool and MVPA [35]. In addition, there was consistent evidence that size of the playground/play area [46, 50], quality of the outdoor environment [50], and convenience of play spaces [42] were positively associated with varied PA. Furthermore, two reviews (both reporting on two studies) examined the association between outdoor balls or play objects and PA, of which one review found evidence for a positive association with TPA [35], and one review found mixed evidence for varied PA [46].

For all other physical environment variables either consistently no evidence for an association (i.e., month, play equipment at home, TV in home, equipment, sedentary items, indoor environments, preschool location, and riding toys) or inconsistent evidence (i.e., time outdoors/in play spaces, attending childcare, season, time of the week, time of the day, weather, portable equipment, teacher (recess) supervision, playground markings, natural features/surfaces, gradients, fixed equipment, preschool type, field trips, electronic media, and playground surfaces with green vegetation) was found across all PA outcome measures.

##### Psychological, Cognitive, and Emotional Variables

Three psychological, cognitive, and/or emotional variables were identified. Two reviews included studies that examined correlates in this domain that showed either no evidence for an association (i.e., knowledge of child), or mixed evidence (i.e., psychosocial health, and cognitive development) [36, 49].

### Consultation of International Researchers

#### First-Round Expert Panel

The consulted international researchers were fairly experienced: of the 31 respondents, 15 indicated that they had more than 10 years of experience in the field of PA in young children. The other 16 respondents indicated having between 5 and 10 years of experience. Eleven respondents were from Australia, ten from North America, nine from Europe, and one from South Africa.

When asked for important determinants that were not on the list of potential correlates derived from the literature, 20 out of 31 researchers added new variables, spread across all five categories: demographic/biological (3), behavioral attributes/skills (6), socio/cultural/policy (20), physical environmental (14), psychological/cognitive/emotional (13) and determinants classified by respondents in the ‘other’ category (5) (i.e., nutrition status, nutrition quality, perceived safety of indoor spaces, dog ownership, and laterality). The majority of the added variables were overlapping with potential correlates identified in the umbrella review (e.g., other wording for a similar construct). The following variables were added by two or more researchers: temperament/personality (4), enjoyment (3), diet/nutrition (3), media use/use of applications (3), perceived motor (skill) competence (3), active travel (2), and perceived outdoor space/neighborhood safety (2).

When asked to select a maximum of ten determinants they considered as most important, participating researchers selected a total of 65 individual determinants: 22 were selected by one researcher, 11 were selected by two researchers, five were selected by three researchers, three were selected by four researchers, and 24 were selected by five or more researchers. Of the determinants that were selected by five or more researchers, nine were social/cultural/policy-related, five were demographic/biological, five were behavioral attributes/skills, and five were physical environmental variables. None of the determinants selected by five or more researchers fell in the category psychological/cognitive/emotional. Table 5 provides an overview of variables that were considered as important determinants of young children’s PA by researchers participating in our expert panel, as well as variables with consistent evidence for an association with PA in our umbrella review.

**Table 5:** Overview of variables that were considered as important determinants of young children’s physical activity (PA) by international researchers, and variables with consistent evidence for an association with PA in our umbrella review. Variables that emerged from both the literature and expert panel are indicated in bold

	Variables considered as ‘most important’ determinants by five or more researchers	Variables with consistent evidence for an association with PA based on at least two reviews (*specific PA outcome)*	Variables with consistent evidence for an association with PA based on one review *(specific PA outcome)*
***Demographic/biological***			
**Sex**	X	X *(MVPA, varied PA*^a^)	
Parental education	X		
SES	X		
Adiposity/BMI	X		
Age	X		
Fitness			X (*TPA)*
Bone/skeletal health			X (*TPA, MVPA)*
***Behavioral attributes/skills***			
**Gross motor skills**	X	X *(MVPA, varied PA)*	
TV viewing/other sedentary behavior	X		
Prone sleeping			X *(varied PA)*
Sleep quality			X *(TPA)*
***Socio/cultural/policy***			
**Parents’ PA/PA role-modelling**	X	X *(varied PA)*^b^	
**Opportunities for play**	X	X *(varied PA)*	
**Parental support**	X	X *(varied PA)*^c^	
**TV viewing rules**	X		X *(varied PA)*
**ECE teacher PA promoting practices**	X		X *(varied PA)*
Time spent playing with peers	X		
Parent perceptions	X		
Parental parenting practices	X		
Parent motivation	X		
Time playing outside with adults	X		
Parental encouragement	X		
ECE PA policy	X		
ECE education confidence and enjoyment	X		
Parental monitoring			X *(varied PA)*
Parent social support			X *(varied PA)*
(Perceived) PA competence			X *(MVPA, varied PA)*
Time spent playing with parent			X *(TPA, varied PA)*
Play rules			X *(TPA, varied PA)*
ECE teacher education/training			X *(MVPA)*
Additional providers at ECE			X *(varied PA)*
***Physical environmental***			
**Outdoor environments**	X		X *(varied PA)*
**Convenience of play space**	X		X (*varied PA)*
Time outdoors/in play spaces	X		
Weather	X		
Size of play area/playground		X *(varied PA)*	
Individual preschool			X *(TPA, varied PA)*
Outdoor balls and play object			X *(TPA)*

*ECE* early childhood education, *MVPA* moderate-to-vigorous physical activity, *PA* physical activity, *SES* socio-economic status, *TPA* total physical activity

^a^Varied PA: when reviews compiled data across multiple quantitative PA outcome measures, we listed these results as “varied PA”. Also, when reviews reported results of PA outcome measures other than LPA, MVPA or TPA (e.g., tummy time), we listed these results as “varied PA”

^b^Association supported by meta-analysis showing a small effect (*r* = 0.1–0.29)[31]

^c^Association supported by meta-analysis showing a medium effect (*r* = 0.3–0.49)[31]

#### Second-Round Expert Panel

In the second round of the expert panel, we presented the outcomes from the first round and a synthesis of findings from the umbrella review (Table 5) to the researchers and asked them to reflect on these outcomes and share their ideas for future research directions.

In summary, consulted researchers reported the following discrepancies between the outcomes of the umbrella review and researcher responses:

- While researchers frequently considered demographic variables (e.g., socioeconomic status, age, BMI) as important determinants of young children’s PA, most of these variables were not identified as correlates in the umbrella review;
- While researchers frequently considered weather and time spent outdoors as important determinants of young children’s PA, these variables were not identified as correlates in the umbrella review;
- While researchers considered multiple parental variables as important determinants of young children’s PA, there was little overlap with the parental correlates identified in the umbrella review;
- While researchers considered peer influence as an important determinant of young children’s PA, this was not identified as a correlate in the umbrella review.

In addition, researchers noticed the relatively small number of variables that were confirmed as a correlate in two or more reviews, and suggested that this hints at the complexity of determinants in this age group.

In general, discrepancies were explained by a broader view of the ‘changing field’ that researchers might have, for example, researchers seem to be more aware of all different factors that may play a role in the development of PA, which may not have been reflected or accounted for in published reviews.

When asked for the topics that need to be addressed in future research, researchers most often mentioned understudied determinants (21 times) and measurement instruments (17 times), followed by the definition of PA (ten times), research design (ten times), and data analysis (seven times). Additionally, researchers mentioned three other important topics for future research (all mentioned by at least two researchers):

- Addressing equity/diversity/disparity/inclusion;
- Applying a more holistic/systemic approach;
- More attention to interventions.

The ranking of these topics confirmed the priority researchers would give to understudied determinants and measurement instruments. In addition, researchers frequently prioritized aforementioned topics that were added by the expert panel.

## Discussion

This systematic umbrella review provides a detailed overview of findings from all available reviews regarding the correlates of PA in children aged 0–5 years. In total, 21 reviews were included that examined 98 different potential correlates. When synthesizing all reviews, 23 correlates were found with consistent evidence for an association with a PA outcome. Notable is the inconsistent evidence across reviews for associations between potential correlates and PA in young children. Although there was little overlap between the correlates identified in the umbrella review and determinants suggested by the expert panel, both confirmed the importance of socio-cultural and policy (e.g., parents, ECE), as well as environmental factors (e.g., outdoor environment, play spaces) in general.

There are several potential explanations for the inconsistencies across reviews that can be considered. First, inconsistencies could be explained by the characteristics of the study populations included. For example, reviews frequently focused on various age groups (i.e., infants, toddlers, and preschoolers), without separately providing results for each age group. However, duration, frequency, intensity, and type of PA are different for children in these age groups, depending on their developmental stage, for example, crawling, walking, and running [11, 53]. Moreover, potential correlates could change dramatically for children from 0 to 5 years of age. Consequently, it is likely that correlates of PA differ between age groups, resulting in mixed results when all age groups are compiled [29]. Additionally, correlates of PA might be different for girls versus boys [54]. Unfortunately, correlates of PA in young children have rarely been examined separately for girls and boys. In line with this, other subgroup differences (e.g., based on cultural differences) between correlates might also result in inconsistencies across reviews. Hence, there may be additional confounding or moderating variables that need to be accounted for in analyses [55]. Unfortunately, discussion of moderators or confounders was rarely provided in the reviews. Importantly, most reviews included studies examining direct and linear associations between single variables and PA [55]. None of the reviews reported on the interrelatedness between correlates of PA, thereby not considering the more recent holistic views on behavior that acknowledge the interrelatedness of variables as part of a dynamic system [56]. Within the ECE environment, for instance, environmental correlates of PA are known to interact with child characteristics and other environmental factors in determining PA [57]. Disregarding the interrelatedness of correlates might lead to false conclusions on an incomplete picture [57].

Second, inconsistencies may be explained by the suboptimal study designs included in the reviews (e.g., cross-sectional studies). In general, there is a lack of studies with a longitudinal or experimental design. Furthermore, only a few reviews have taken differences in study designs into account in their reporting or analyses [36, 39, 43]. Some of the variables with consistent evidence for an association with PA have only been studied cross-sectionally and thus the direction of the relationship is less apparent (e.g., sleep quality, prone sleeping, fitness, and bone health). Longitudinal study designs are necessary to disentangle cause and consequences, as well as potential bi-directionality of such relationships. Sufficient sleep, for instance, has been shown to be both a cause and a consequence of increased PA in adults [58] and older children [59], which might also be the case for young children. The expert panel underlined the importance of longitudinal and intervention designs in future research.

Third, the majority of the reviews compiled the results of a variety of PA outcome measures (e.g., different PA intensities and types of PA). However, associations with PA may differ per PA outcome measure [36, 39]. The heterogeneity of PA outcome measures across the studies included in the reviews makes it impossible to consistently analyze these outcomes separately. In addition, PA outcome measures that are commonly used in studies of adults and older children (e.g., LPA and MVPA) are frequently used in PA research in early childhood. Since infants, toddlers, and preschoolers each have their own form and context of PA [3], tailored PA outcome measures are needed. Consequently, caution is required when interpreting results of different PA intensities in this age group. Unfortunately, guidelines on how PA should be defined in infants, toddlers, and preschoolers, and how these subsequently should be assessed, are lacking, and are urgently needed [3, 49]. Our expert panel indeed emphasized this need.

Similarly, more detail on the definition of correlates is needed. We were unable to interpret various findings of the included reviews because of a lack of clearly defined variables (e.g., ‘play rules’ or ‘child monitoring’), sometimes also lacking the context (e.g., whether correlates referred to the home or ECE environment). Furthermore, assessment of correlates was often unclear (e.g., instrument used) or constructs were sometimes overlapping, especially between various parental constructs (e.g., between parental support, parental social support, parental encouragement). Consensus on conceptualization and measurement of PA parenting has previously been indicated as a priority as well [60]. It must be noted, however, that we did not go back to individual studies. Therefore, we cannot confirm whether details regarding (the examination of) correlates were also lacking in the studies included in reviews.

Last, the lack of appropriate measurement instruments makes it even more difficult to assess PA in young children. Available measurement instruments, both direct (e.g., accelerometer) and indirect (e.g., questionnaire), all have substantial limitations and generally have unknown or insufficient validity and reliability in this age group [61–63]. Although accelerometry is widely considered the most promising method for PA assessment, validated methods in children up to the age of 3 years are currently lacking [62]. This has a major impact on the quality of the studies and the validity of the results. Until accurate measurement instruments for all developmental stages are available, caution is needed when interpreting PA results in this young age group. Similarly, measurement instruments to assess correlates of PA often have unknown or insufficient measurement properties [60, 64, 65]. Consequently, some variables might be understudied because they are difficult to measure. For example, as mentioned earlier, our umbrella review indicates that psychological, cognitive, and/or emotional correlates have rarely been studied, which is probably due to the difficulty of measuring these factors in young children [66].

### Evidence for Correlates of Physical Activity

While we cannot draw any firm conclusions, we found consistent evidence for some correlates of PA in young children. With regard to the demographic and biological variables, the majority of reviews found that generally boys are more active than girls [35, 41, 42, 50], which is consistent with results in older children and adolescents [29]. Next to male sex, our expert panel also frequently mentioned age as an important determinant of increased PA. However, our umbrella review found inconsistent evidence for age, which could partially be explained by the different age ranges in the reviews. While overall, age might not be a correlate of PA, within specific age groups (e.g., infants) age might be a correlate of PA [37]. This suggests that the positive association between age and PA might be non-linear especially in young children, which fits the dynamic systems theory in which (motor) development of young children is seen as a non-linear and discontinuous process [53, 67–69]. As the transition to primary school is associated with decreased PA levels in children [70], the potential influence of the primary school environment on PA might also account for some of the mixed results for age [71, 72]. This may be due to the fact that in some countries children aged 4 years already attend primary school, while in others children start later [73].

With regard to the category behavioral attributes and skills, young children’s motor skills were positively associated with PA in most reviews, especially for higher PA intensities (i.e., MVPA) [35, 36, 49, 51]. Based on three cross-sectional studies, no evidence for an association was found between motor skills and LPA [49]. However, as mentioned earlier, caution is needed when interpreting results of different PA intensities in this age group. Current literature also showed cross-sectional associations between PA and sleep quality in toddlers [52] and prone sleeping in infants [37], although these were not suggested as important determinants by our expert panel.

We found consistent evidence for several socio-cultural and policy variables as correlates of PA. For example, parental practices such as parental role modeling [33, 36, 42–44], parental support [33, 44], parental monitoring [36], and time spent playing with parents [39, 43] were positively associated with young children's PA. Moreover, there was consistent evidence for rule setting as a correlate of PA behavior, with rules for watching TV having a positive association and playing rules having a negative association with PA levels [35, 42]. Evidence for a positive association was also seen with opportunities for play [36, 50]. At the ECE level, promoting PA by PA teachers [47], as well as other ECE staff [36], might also increase PA. In general, both the literature and our expert panel confirmed the importance of the social environment in young children’s PA.

Several physical environmental variables have consistently shown an association with increased PA levels in young children. These include the individual preschool [39, 42], size of playground area [46, 50], availability of outdoor balls and other play objects [35], presence of outdoor environments [50], and convenience of play spaces [42]. These findings suggest that the availability of play areas and play opportunities may have an impact on PA in early childhood, and should therefore be considered within policies of ECE and neighborhood design, as well as communicated to parents.

### Gaps in Literature and Recommendations for Future Studies

We propose a number of recommendations for future studies based on our umbrella review and the consultation of international researchers. First, our expert panel prioritized understudied determinants as direction for future research. There are multiple factors that might be relevant for young children’s PA that do not (yet) appear in systematic reviews. Researchers participating in the expert panel suggested several potential determinants that need further investigation, such as gaming and other sedentary behaviors, peer influence, parental variables, and the public environment.

Secondly, most studies were conducted within high-income countries in North America and Europe, with little research conducted in low- and middle-income countries. As confirmed by the expert panel, future studies need to address aspects with regard to diversity, equity, and disparity, for example by using socio-culturally sensitive research methods [74]. Moreover, there is a dearth of studies for 0- to 2-year-olds. Although multiple reviews included children across the entire age range (0–5.9 years old), studies examining PA in toddlers and/or infants are rare. Hence, future studies aimed at examining the youngest age groups are urgently needed.

Thirdly, future studies should focus on developing and improving measurement instruments for assessing PA as well as correlates in 0- to 5-year-olds, a need supported by our expert panel. Subsequently, when appropriate measurement instruments are available, we recommend future studies to use longitudinal or experimental study designs to examine correlates of PA.

In line with the expert panel’s prioritization for future research, we recommend future studies to take a more comprehensive or holistic approach when investigating correlates, taking multifactorial interactions between correlates into account from a systems perspective [55–57, 75]. Although challenging, the analysis and reporting of interactions between potential correlates of PA is recommended to gain insight into the complex interrelations between a wide variety of correlates [75].

Regarding the quality of systematic reviews, we recommend that future reviews carefully apply PRISMA guidelines [24], which are a valuable tool for structuring systematic literature reviews (e.g., report on data-extraction and screening methods, as well as consider publication bias in analyzing/discussing results). In addition, we recommend future reviews to weigh the methodological quality or differentiate between studies of different quality when synthesizing study results. Moreover, it is important that reviews provide clear definitions of the included variables.

### Strengths and Limitations

The current findings need to be considered in light of several strengths and limitations. Strong points of this umbrella review include the duplicate and independent screening, data extraction and quality assessment. In addition, four different databases with varied focuses were searched (i.e., PubMed, Embase, PsycINFO, and SPORTDiscus) to gather a wide range of related literature. Furthermore, we distinguished between age groups and PA outcome measures in reporting our results where possible, providing a thorough understanding of what is known and what gaps still exist in the extensive literature on the topic of PA in young children. We also examined reviews that included cross-sectional studies investigating a potential (reverse) relationship between PA and an outcome, which may identify possible relationships that otherwise would have been missed. On the other hand, including cross-sectional studies is also a limitation as it does not allow us to examine causal relationships. An additional strength was the incorporation of a panel of researchers in this field, enriching and supplementing the conclusions from the umbrella review.

The limitations of the umbrella review include that important information might have been lost while combining conclusions from various studies, due to a lack of details reported in the reviews. It was not feasible to go back to the individual studies included in the reviews to retrieve the information. In addition, we included only reviews written in English and expert panel members who could speak English, disregarding research published in other languages and experts who do not master the English language. Moreover, as experts for the consultation were recruited based on snowball sampling, we may not have included all relevant areas of young children’s PA. Consequently, some potential determinants may not have emerged from the expert panel (e.g., children’s physical literacy [76]). Furthermore, we did not account for overlap of primary studies included in multiple reviews, which may have led to double counting of some of the results, potentially leading to an overestimation or underestimation of the associations found. Another limitation is the date of our literature search (i.e., May 2020), which means that reviews published in the last 2 years are not included in our umbrella review. We decided not to do a search update, as our expert panel was based on the results of our initial search, and an update would mean that findings of the expert panel could not be compared to the literature search. As a result, the findings of our umbrella review need to be interpreted with caution, since developments that have taken place in the most recent years are most likely to be underrepresented in our review. This shortcoming is underlined by the few studies on screen time included in published reviews. Finally, while the quality assessment allowed for the identification of methodological strengths and shortcomings in the literature as well as the development of specific recommendations for future research, we did not quantify these quality measures and take the quality ratings into account when weighing the evidence due to the large heterogeneity of the included reviews, which can be seen as a limitation.

## Conclusion

Multiple correlates of PA in 0- to 5-year-old children were identified. However, various methodological challenges (e.g., measurement instruments) and the large heterogeneity (e.g., study samples, correlates, and outcome measures) hindered formulating clear conclusions. Moreover, none of the reviews reported on the interrelatedness between correlates, which would correspond with more holistic views on behavior. These findings indicate the urgent need for establishing a common ground in definitions, assessment methods, and analytical methods to further the field of PA research in this tremendously important age group.

## Supplementary Information

Below is the link to the electronic supplementary material.

Supplementary file1 (PDF 167 KB)

## Declarations

### Availability of data and materials

The data that support the findings of this review are available from the corresponding author upon reasonable request.
