# Supplementary material for: Correlates of Physical Activity in 0- to 5-year-olds: A Systematic Umbrella Review and Consultation of International Researchers
Source: Sports Med. 2022 Oct 11;53(1):215–40. doi: 10.1007/s40279-022-01761-5 (PMC9807466; doi:10.1007/s40279-022-01761-5)
Supplement: Supplementary file 1 — Supplementary file1 (PDF 167 KB) [file 40279_2022_1761_MOESM1_ESM.pdf]

Correlates of Physical Activity in 0- to 5-year-olds: A Systematic Umbrella Review and Consultation of International Researchers

Jelle Arts[1] *, Elizabeth Drotos[2] *, Amika S. Singh[3,4], Mai J. M. Chinapaw[1], Teatske M. Altenburg[1], Jessica S. Gubbels[2]

[1] Department of Public and Occupational Health, Amsterdam Public Health, Amsterdam UMC, Vrije Universiteit Amsterdam, De Boelelaan 1117, Amsterdam, The Netherlands;

[2] Department of Health Promotion, NUTRIM School of Nutrition and Translational Research in Metabolism, Maastricht University, Maastricht, The Netherlands

[3] Mulier Institute, Utrecht, The Netherlands;

[4] Center for Physically Active Learning, Faculty of Education, Arts and Sports, Western Norway University of Applied Sciences, Sogndal, Norway.

* These authors contributed equally to this work

Journal:

Sports Medicine

Corresponding author:

Jelle Arts

j.arts@amsterdamumc.nl

Online Resource 1: Search terms systematic umbrella review

Search done: 4 May 2020

Population

Pubmed

"Infant"[Mesh:noexp] OR "Infant, Newborn"[Mesh:noexp] OR "Child, Preschool"[Mesh] OR infan*[tiab] OR newborn*[tiab] OR new-born*[tiab] OR neonate*[tiab] OR baby[tiab] OR babies[tiab] OR toddler*[tiab] OR preschool*[tiab] OR pre-school*[tiab] OR kindergarten*[tiab] OR childcare*[tiab] OR daycare*[tiab] OR nurser*[tiab] OR ECEC[tiab] OR early childhood[tiab] OR early years[tiab] OR early life[tiab] OR children[tiab]

Embase

exp infant/ OR exp baby/ OR exp newborn/ OR exp preschool child/ OR exp toddler/ OR exp early childhood/ OR exp early life/ OR infan*.ab,ti,kw. OR newborn*.ab,ti,kw. OR new-born*.ab,ti,kw. OR neonate*.ab,ti,kw. OR baby.ab,ti,kw. OR babies.ab,ti,kw. OR toddler*.ab,ti,kw. OR preschool*.ab,ti,kw. OR pre-school*.ab,ti,kw. OR kindergarten*.ab,ti,kw. OR childcare*.ab,ti,kw. OR daycare*.ab,ti,kw. OR nurser*.ab,ti,kw. OR ECEC.ab,ti,kw. OR "early childhood".ab,ti,kw. OR "early years".ab,ti,kw. OR early life.ab,ti,kw. OR children.ab,ti,kw.

PsycINFO

DE "Early Childhood Development" OR DE "Infant Development" OR DE "Neonatal Development" OR DE "Preschool Students" OR DE "Nursery School Students" OR DE "Kindergarten Students" OR TI (infan* OR newborn* OR "new-born*" OR neonate* OR baby OR babies OR toddler* OR preschool* OR "pre-school*" OR kindergarten* OR childcare* OR daycare* OR nurser* OR ECEC OR "early childhood" OR "early years" OR "early life" OR children) OR AB (infan* OR newborn* OR "new-born*" OR neonate* OR baby OR babies OR toddler* OR preschool* OR "pre-school*" OR kindergarten* OR childcare* OR daycare* OR nurser* OR ECEC OR "early childhood" OR "early years" OR "early life" OR children) OR KW (infan* OR newborn* OR "new-born*" OR neonate* OR baby OR babies OR toddler* OR preschool* OR "pre-school*" OR kindergarten* OR childcare* OR daycare* OR nurser* OR ECEC OR "early childhood" OR "early years" OR "early life" OR children)

SportDiscus

TI (infan* OR newborn* OR "new-born*" OR neonate* OR baby OR babies OR toddler* OR preschool* OR "pre-school*" OR kindergarten* OR childcare* OR daycare* OR nurser* OR ECEC OR "early childhood" OR "early years" OR "early life" OR children) OR AB (infan* OR newborn* OR "new-born*" OR neonate* OR baby OR babies OR toddler* OR preschool* OR "pre-school*" OR kindergarten* OR childcare* OR daycare* OR nurser* OR ECEC OR "early childhood" OR "early years" OR "early life" OR children) OR KW (infan* OR newborn* OR "new-born*" OR neonate* OR baby OR babies OR toddler* OR preschool* OR "pre-school*" OR kindergarten* OR childcare* OR daycare* OR nurser* OR ECEC OR "early childhood" OR "early years" OR "early life" OR children)

Physical Activity

Pubmed

"Motor Activity"[Mesh:NoExp] OR "Exercise"[Mesh] OR "Sports"[Mesh] OR motor activit*[tiab] OR physical activit*[tiab] OR locomotor activit*[tiab] OR exercis*[tiab] OR running[tiab] OR run[tiab] or sport*[tiab] OR active[tiab] OR walk*[tiab] OR outdoor[tiab] OR play*[tiab] OR bicycle[tiab] OR cycle[tiab] OR bicycling[tiab] OR cycling[tiab] OR biking[tiab] OR "tummy time"[tiab] OR "floor time"[tiab] OR "prone

position"[tiab] OR crawl*[tiab] OR swim*[tiab] OR "rough and tumble"[tiab] OR "Movement"[MeSH:NoExp] OR movement[tiab]

Embase

exp 'motor activity'/ OR exp 'exercise'/ OR exp 'sport'/ OR exp "movement (physiology)"/ OR "motor activit*".ab,ti,kw. OR "physical activit*".ab,ti,kw. OR "locomotor activit*".ab,ti,kw. OR exercis*.ab,ti,kw. OR running.ab,ti,kw. OR run.ab,ti,kw. OR sport*.ab,ti,kw. OR active.ab,ti,kw. OR walk*.ab,ti,kw. OR outdoor.ab,ti,kw. OR play*.ab,ti,kw. OR bicycle.ab,ti,kw. OR cycle.ab,ti,kw. OR bicycling.ab,ti,kw. OR cycling.ab,ti,kw. OR biking.ab,ti,kw. OR "tummy time".ab,ti,kw. OR "floor time".ab,ti,kw. OR "prone position".ab,ti,kw. OR crawl*.ab,ti,kw. OR swim*.ab,ti,kw. OR "rough adj2 tumble".ab,ti,kw. OR movement.ab,ti,kw.

PsycINFO

DE "HUMAN locomotion" OR DE RUNNING OR DE SWIMMING OR DE WALKING OR DE CYCLING OR SU("PHYSICAL activity" OR "BODY **movement**" OR "PHYSICAL **fitness**" OR "**EXERCISE**") OR TI("motor activit*" OR "physical activit*" OR "locomotor activit*" OR exercis* OR running OR run OR sport* OR active OR walk* OR outdoor OR play* OR bicycle OR cycle OR bicycling OR cycling OR biking OR "tummy time" OR "floor time" OR "prone position" OR crawl* OR swim* OR "rough and tumble" OR movement) OR AB("motor activit*" OR "physical activit*" OR "locomotor activit*" OR exercis* OR running OR run OR sport* OR active OR walk* OR outdoor OR play* OR bicycle OR cycle OR bicycling OR cycling OR biking OR "tummy time" OR "floor time" OR "prone position" OR crawl*OR swim*OR "rough and tumble" OR movement) OR KW("motor activit*" OR "physical activit*" OR "locomotor activit*" OR exercis* OR running OR run OR sport* OR active OR walk* OR outdoor OR play* OR bicycle OR cycle OR bicycling OR cycling OR biking OR "tummy time" OR "floor time" OR "prone position" OR crawl* OR swim* OR "rough and tumble" OR movement)

SportDiscus

DE("HUMAN locomotion" OR "RUNNING" OR "SWIMMING" OR "WALKING" OR "CYCLING") OR SU("PHYSICAL activity" OR "BODY **movement**" OR "PHYSICAL **fitness**" OR "**EXERCISE**") OR TI("motor activit*" OR "physical activit*" OR "locomotor activit*" OR exercis* OR running OR run OR sport* OR active OR walk* OR outdoor OR play* OR bicycle OR cycle OR bicycling OR cycling OR biking OR "tummy time" OR "floor time" OR "prone position" OR crawl* OR swim* OR "rough and tumble" OR movement) OR AB("motor activit*" OR "physical activit*" OR "locomotor activit*" OR exercis* OR running OR run OR sport* OR active OR walk* OR outdoor OR play* OR bicycle OR cycle OR bicycling OR cycling OR biking OR "tummy time" OR "floor time" OR "prone position" OR crawl*OR swim*OR "rough and tumble" OR movement) OR KW("motor activit*" OR "physical activit*" OR "locomotor activit*" OR exercis* OR running OR run OR sport* OR active OR walk* OR outdoor OR play* OR bicycle OR cycle OR bicycling OR cycling OR biking OR "tummy time" OR "floor time" OR "prone position" OR crawl* OR swim* OR "rough and tumble" OR movement)

<u>Determinants/Correlates</u>

Pubmed

regression[tiab] OR predicted[tiab] OR relation[tiab] OR relationship*[tiab] OR related[tiab] OR associated[tiab] OR association*[tiab] OR correlat*[ti] OR determinant*[ti] OR risk factor*[ti] OR correlat*[ot] OR determinant*[ot] OR risk factor*[ot]

Embase

regression.ab,ti,kw. OR predicted.ab,ti,kw. OR relation.ab,ti,kw. OR relationship*.ab,ti,kw. OR related.ab,ti,kw. OR associated.ab,ti,kw. OR association*.ab,ti,kw. OR correlat*.ti. OR determinant*.ti. OR "risk factor*".ti. OR correlat*.kw. OR determinant*.kw. OR "risk factor*".kw

PsycINFO

TI (regression OR predicted OR relation OR relationship* OR related OR associated OR association* OR correlat* OR determinant* OR "risk factor*") OR AB (regression OR predicted OR relation OR relationship* OR related OR associated OR association*) OR KW (regression OR predicted OR relation OR relationship* OR related OR associated OR association* OR correlat* OR determinant* OR "risk factor*")

SportDiscus

TI (regression OR predicted OR relation OR relationship* OR related OR associated OR association* OR correlat* OR determinant* OR "risk factor*") OR AB (regression OR predicted OR relation OR relationship* OR related OR associated OR association*) OR KW (regression OR predicted OR relation OR relationship* OR related OR associated OR association* OR correlat* OR determinant* OR "risk factor*")

<u>Review</u>

Pubmed

systematic review[tiab] OR literature review[tiab] OR scoping review[tiab] OR narrative review[tiab] OR meta-analysis[tiab] OR meta analysis[tiab]

Embase

"systematic review".ab,ti,kw. OR "literature review".ab,ti,kw. OR "scoping review".ab,ti,kw. OR "narrative review".ab,ti,kw. OR "meta-analysis".ab,ti,kw. OR "meta analysis".ab,ti,kw.

PsycINFO

TI (“systematic review” OR” literature review” OR “scoping review” OR “narrative review” OR “meta-analysis” OR “meta analysis”)

SportDiscus

TI (“systematic review” OR” literature review” OR “scoping review” OR “narrative review” OR “meta-analysis” OR “meta analysis”)

<u>Potential ‘NOT’ terms</u>

Pubmed

NOT ("Diseases Category"[Majr] OR "Attention Deficit and Disruptive Behavior Disorders"[Majr] OR "Child Development Disorders, Pervasive"[Majr])

NOT ("Animals"[Mesh] NOT "Humans"[Mesh])

Embase

NOT (exp diseases/ or exp "attention deficit disorder"/ or exp autism/)

NOT ([animals]/lim NOT [humans]/lim)

NOT ("conference abstract" or "conference review").dj.

PsycINFO

NOT (MM "Disorders" OR MM "Disabilities" OR MM "Lesions" OR MM "Adventitious Disorders" OR MM "Atypical Disorders" OR MM "Behavior Disorders" OR MM "Chronic Illness" OR MM "Communication Disorders" OR MM "Congenital Disorders" OR MM "Disorder Attributes" OR MM "Mental Disorders" OR MM "Physical Disorders" OR MM "Sensory Integration Dysfunction" OR MM "Treatment Resistant Disorders"

OR MM "Disability Management" OR MM "Learning Disabilities" OR MM "Multiple Disabilities" OR MM "Reading Disabilities" OR MM "Brain Lesions (Disorders)" OR MM "Brain Lesions (Experimental)" OR MM "Neural Lesions" OR MM "Atypical Depression" OR MM "Conduct Disorder" OR MM "Disruptive Behavior Disorders" OR MM "Homicide" OR MM "Impulse Control Disorders" OR MM "Juvenile Delinquency" OR MM "Kleptomania" OR MM "Oppositional Defiant Disorder" OR MM "Pyromania" OR MM "Self-Destructive Behavior" OR MM "Chronic Alcoholic Intoxication" OR MM "Chronic Fatigue Syndrome" OR MM "Chronic Mental Illness" OR MM "Chronic Pain" OR MM "Chronically Ill Children" OR MM "Hearing Disorders" OR MM "Language Disorders" OR MM "Agenesis" OR MM "Cleft Palate" OR MM "Drug Induced Congenital Disorders" OR MM "Hermaphroditism" OR MM "Microcephaly" OR MM "Prader Willi Syndrome" OR MM "Spina Bifida" OR MM "Chronicity (Disorders)" OR MM "Disease Course" OR MM "Disease Progression" OR MM "Disease Transmission" OR MM "Etiology" OR MM "Onset (Disorders)" OR MM "Predisposition" OR MM "Prognosis" OR MM "Recovery (Disorders)" OR MM "Relapse (Disorders)" OR MM "Remission (Disorders)" OR MM "Sequelae" OR MM "Severity (Disorders)" OR MM "Subtypes (Disorders)" OR MM "Susceptibility (Disorders)" OR MM "Borderline States" OR MM "Thought Disturbances" OR MM "Affective Disorders" OR MM "Anxiety Disorders" OR "Attention Deficit Disorder" OR MM "Attention Deficit Disorder with Hyperactivity" OR MM "Autism Spectrum Disorders" OR MM "Autistic Traits" OR MM "Bipolar Disorder" OR MM "Chronic Mental Illness" OR MM "Dissociative Disorders" OR MM "Eating Disorders" OR MM "Gender Dysphoria" OR MM "Mental Disorders due to General Medical Conditions" OR MM "Neurocognitive Disorders" OR MM "Neurodevelopmental Disorders" OR MM "Neurosis" OR MM "Paraphilias" OR MM "Personality Disorders" OR MM "Psychosis" OR MM "Sleep Wake Disorders" OR MM "Somatoform Disorders" OR MM "Stress and Trauma Related Disorders" OR MM "Substance Related and Addictive Disorders" OR MM "Blood and Lymphatic Disorders" OR MM "Anemia" OR MM "Hemophilia" OR MM "Leukemias" OR MM "Malaria" OR MM "Porphyria" OR MM "Rh Incompatibility" OR MM "Sickle Cell Disease" OR MM "Cachexia" OR MM "Cardiovascular Disorders" OR DE "Aneurysms" OR MM "Arteriosclerosis" OR MM "Atherosclerosis" OR MM "Cerebral Arteriosclerosis" OR MM "Blood Pressure Disorders" OR MM "Hypertension" OR MM "Hypotension" OR MM "Syncope" OR MM "Cerebrovascular Disorders" OR MM "Cerebral Arteriosclerosis" OR MM "Cerebral Hemorrhage" OR MM "Cerebral Ischemia" OR MM "Cerebral Small Vessel Disease" OR MM "Cerebrovascular Accidents" OR MM "Subarachnoid Hemorrhage" OR MM "Embolisms" OR MM "Heart Disorders" OR MM "Angina Pectoris" OR MM "Arrhythmias (Heart)" OR MM "Coronary Thromboses" OR MM "Myocardial Infarctions" OR MM "Hemorrhage" OR MM "Cerebral Hemorrhage" OR MM "Hematoma" OR MM "Subarachnoid Hemorrhage" OR MM "Hypertension" OR MM "Ischemia" OR MM "Cerebral Ischemia" OR MM "Cerebral Small Vessel Disease" OR MM "Thromboses" OR MM "Coronary Thromboses" OR MM "Chronically Ill Children" OR MM "Digestive System Disorders" OR MM "Cystic Fibrosis" OR MM "Dyspepsia" OR MM "Gastrointestinal Disorders" OR MM "Colon Disorders" OR MM "Dysphagia" OR MM "Gastrointestinal Ulcers" OR MM "Vomiting" OR MM "Liver Disorders" OR MM "Cirrhosis (Liver)" OR MM "Hepatitis" OR MM "Jaundice" OR MM "Endocrine Disorders" OR MM "Adrenal Gland Disorders" OR MM "Addisons Disease" OR MM "Cushings Syndrome" OR MM "Diabetes" OR MM "Diabetes Insipidus" OR MM "Diabetes Mellitus" OR MM "Type 2 Diabetes" OR MM "Endocrine Neoplasms" OR MM "Parathyroid Disorders" OR MM "Pituitary Disorders" OR MM "Hypopituitarism" OR MM "Thyroid Disorders" OR MM "Goiters" OR MM "Hyperthyroidism" OR MM "Hypothyroidism" OR MM "Thyrotoxicosis" OR MM "Genetic Disorders" OR MM "Albinism" OR MM "Charcot-Marie-Tooth Disease" OR MM "Chromosome Disorders" OR MM "Cornelia De Lange Syndrome" OR MM "Huntingtons Disease" OR MM "MELAS" OR MM "Neurofibromatosis" OR MM "Phenylketonuria" OR MM "Porphyria" OR MM "Rh Incompatibility" OR MM "Sex Linked Hereditary Disorders" OR MM "Sickle Cell Disease" OR MM "Tay Sachs Disease" OR MM "Williams Syndrome" OR MM "Health Impairments" OR MM "Immunologic Disorders" OR MM "Allergic Disorders" OR MM "Allergic Skin Disorders" OR MM "Drug Allergies" OR MM "Food Allergies" OR MM "Hay Fever" OR MM "Anaphylactic Shock" OR MM "Celiac Disease" OR MM "Guillain-Barre Syndrome" OR MM "HIV" OR MM "AIDS" OR MM "Rh Incompatibility" OR MM "Infectious Disorders" OR MM "Bacterial Disorders" OR MM "Bacterial Meningitis" OR MM "Gonorrhea" OR MM "Lyme Disease" OR MM "Pulmonary Tuberculosis" OR MM "Tuberculosis" OR MM "Intracranial Abscesses" OR MM "Parasitic Disorders" OR MM "Malaria" OR MM "Sexually Transmitted Diseases" OR MM "AIDS" OR MM "Gonorrhea" OR MM "HIV" OR MM "Herpes Genitalis" OR MM "Syphilis" OR MM "Viral Disorders" OR MM "Encephalitis" OR MM "Epstein Barr Viral Disorder" OR MM "HIV" OR MM "Herpes Genitalis" OR MM "Herpes Simplex" OR MM "Human Papillomavirus" OR MM "Influenza" OR MM "Measles" OR MM "Poliomyelitis" OR MM "Rubella" OR MM "Metabolism Disorders" OR MM "Hyperglycemia" OR MM "Hypoglycemia" OR MM "Hyponatremia" OR

MM "Lipid Metabolism Disorders" OR MM "Phenylketonuria" OR MM "Porphyria" OR MM "Musculoskeletal Disorders" OR MM "Bone Disorders" OR MM "Osteoporosis" OR MM "Joint Disorders" OR MM "Arthritis" OR MM "Rheumatic Fever" OR MM "Muscular Disorders" OR MM "Cataplexy" OR MM "Fibromyalgia" OR MM "Muscular Atrophy" OR MM "Muscular Dystrophy" OR MM "Myasthenia Gravis" OR MM "Myofascial Pain" OR MM "Myotonia" OR MM "Torticollis" OR MM "Neonatal Disorders" OR MM "Anencephaly" OR MM "Cleft Palate" OR MM "Conjoined Twins" OR MM "Crying Cat Syndrome" OR MM "Down's Syndrome" OR MM "Klinefelters Syndrome" OR MM "Phenylketonuria" OR MM "Tay Sachs Disease" OR MM "Turners Syndrome" OR MM "Neoplasms" OR MM "Benign Neoplasms" OR MM "Breast Neoplasms" OR MM "Endocrine Neoplasms" OR MM "Leukemias" OR MM "Melanoma" OR MM "Metastasis" OR MM "Brain Neoplasms" OR MM "Glioma" OR MM "Nervous System Disorders" OR MM "Autonomic Nervous System Disorders" OR MM "Central Nervous System Disorders" OR MM "Brain Disorders" OR MM "Chorea" OR MM "Meningitis" OR MM "Myelitis" OR MM "Anencephaly" OR MM "Athetosis" OR MM "Cerebral Atrophy" OR MM "Cerebrovascular Accidents" OR MM "Diaschisis" OR MM "Encephalitis" OR MM "Encephalopathies" OR MM "Epilepsy" OR MM "Epileptic Seizures" OR MM "General Paresis" OR MM "Hydrocephalus" OR MM "Leukoencephalopathy" OR MM "Microcephaly" OR MM "Parkinson's Disease" OR MM "Movement Disorders" OR MM "Alien Limb Syndrome" OR MM "Apraxia" OR MM "Ataxia" OR MM "Athetosis" OR MM "Catalepsy" OR MM "Cataplexy" OR MM "Dyskinesia" OR MM "Dyspraxia" OR MM "Paralysis" OR MM "Spasms" OR MM "Muscle Spasms" OR MM "Tremor" OR MM "Hemiparesis" OR MM "Hemiplegia" OR MM "Paraplegia" OR MM "Quadriplegia" OR MM "Cerebral Palsy" OR MM "Nervous System Neoplasms" OR MM "Neurodegenerative Diseases" OR MM "Neurofibromatosis" OR MM "Neuroinflammation" OR MM "Neuromuscular Disorders" OR MM "Myopathy" OR MM "Neuropathy" OR MM "Perceptual Disturbances" OR MM "Agnosia" OR MM "Sensory Neglect" OR MM "Sclerosis (Nervous System)" OR MM "Multiple Sclerosis" OR MM "Seizures" OR MM "Nutritional Deficiencies" OR MM "Protein Deficiency Disorders" OR MM "Kwashiorkor" OR MM "Vitamin Deficiency Disorders" OR MM "Pellagra" OR MM "Wernicke's Syndrome" OR MM "Physical Disfigurement" OR MM "Respiratory Tract Disorders" OR MM "Apnea" OR MM "Sleep Apnea" OR MM "Bronchial Disorders" OR MM "Dyspnea" OR MM "Asthma" OR MM "Hyperventilation" OR MM "Laryngeal Disorders" OR MM "Lung Disorders" OR MM "Chronic Obstructive Pulmonary Disease" OR MM "Cystic Fibrosis" OR MM "Pneumonia" OR MM "Pulmonary Emphysema" OR MM "Pulmonary Tuberculosis" OR MM "Pharyngeal Disorders" OR MM "Sense Organ Disorders" OR MM "Anosmia" OR MM "Ear Disorders" OR MM "Labyrinth Disorders" OR MM "Tinnitus" OR MM "Taste Disorders" OR MM "Vision Disorders" OR MM "Balint's Syndrome" OR MM "Blind" OR MM "Blindsight" OR MM "Eye Disorders" OR MM "Hemianopia" OR MM "Partially Sighted" OR MM "Sensory System Disorders" OR MM "Somatosensory Disorders" OR MM "Skin Disorders" OR MM "Allergic Skin Disorders" OR MM "Alopecia" OR MM "Dermatitis" OR MM "Herpes Simplex" OR MM "Lupus" OR MM "Pruritus" OR MM "Toxic Disorders" OR MM "Urogenital Disorders" OR MM "Genital Disorders" OR MM "Gynecological Disorders" OR MM "Kidney Diseases" OR MM "Urinary Function Disorders" OR MM "Urinary Incontinence" OR MM "Vision Disorders" OR MM "Balint's Syndrome" OR MM "Blind" OR MM "Deaf Blind" OR MM "Blindsight" OR MM "Eye Disorders" OR MM "Amblyopia" OR MM "Cataracts" OR MM "Color Blindness" OR MM "Glaucoma" OR MM "Nystagmus" OR MM "Refraction Errors" OR MM "Strabismus" OR MM "Tunnel Vision" OR MM "Hemianopia" OR MM "Partially Sighted" OR MM "Work Related Illnesses" OR MM "Treatment Resistant Depression" OR (TI ("cerebral palsy" OR autism OR autistic OR "attention deficit" OR ADHD) OR KW ("cerebral palsy" OR autism OR autistic OR "attention deficit" OR ADHD))

NOT (PO Animal NOT PO Human)

SportDiscus

NOT (MM "CEREBRAL palsy" OR MM "ATTENTION-deficit hyperactivity disorder" OR MM "ATTENTION-deficit-disordered children" OR TI ("cerebral palsy" OR autism OR autistic OR "attention deficit" OR ADHD) OR KW ("cerebral palsy" OR autism OR autistic OR "attention deficit" OR ADHD))

Online resource 2: Adapted version of the modified AMSTAR tool developed by Pollock et al. (2014) [1, 2].

	**Item**	**Guidance from the AMSTAR**	**Additional questions to note (requires a yes on all sub-questions)**	**Answer options**
1	Was an 'a priori' design provided?	The research question and inclusion criteria should be established before the conduct of the review	Were review 1) populations 2) interventions OR exposures 3) and outcomes specified?	Yes / No / Unclear
2a	Was there duplicate study selection and data extraction?	There should be at least two independent data extractors and a consensus procedure for disagreements should be in place.	Were studies 1) assessed for inclusion by 2 independent reviewers with a consensus procedure? [use "partial" when only a portion is independently performed]	Yes / No / Partial / Unclear
2b			AND 2) data extracted by 2 independent reviewers with a consensus procedure? [use "partial" when only a portion is independently performed]	Yes / No / Partial / Unclear
3a	Was a comprehensive literature search performed?	At least two electronic sources should be searched. The report must include years and databases used (e.g., EMBASE). Keywords and/or MESH terms must be stated and where feasible the search strategy should be provided. All searches should be supplemented by consulting current contents, reviews, textbooks, specialized registers, or experts in the particular field of study, and by reviewing the references in the studies found.	Clarification note: For any one of the listed supplementary searches would get a 'yes.'	Yes / No / Unclear
3b			The report must include years searched.	Yes / No

4a	Was the status of publication (i.e. grey literature) used as an inclusion criterion?	The authors should state that they searched for reports regardless of their publication type. The authors should state whether or not they excluded any reports (from the systematic review), based on their publication status, language etc.	Were no filters used? If there is no specific statement, enter ’unclear’	Yes / No / Unclear
4b			Is it clear how language and publication type are used in eligibility criteria to exclude reports?	Yes / No / Unclear
5	Was a list of studies (included and excluded) provided?	A list of included and excluded studies should be provided.	Is there a flow diagram included with reasons for exclusion? (Or narrative explanation of what kinds of studies were excluded)	Yes / No / Unclear
6a	Were the characteristics of the included studies provided?	In an aggregated form such as a table, data from the original studies should be provided on the participants, interventions and outcomes. The ranges of characteristics in all the studies analyzed e.g., age, race, sex, relevant socioeconomic data, disease status, duration, severity, or other diseases should be reported.	1) Were details provided on the **participants** of included studies (including age, gender, relevant information related to socioeconomic status or race, overweight status)?	Yes / No / Unclear
6b			2) Were details provided on the **exposure/factors**? Including how possible correlates were measured?	Yes / No / Unclear
6c			3) Were details provided on the outcomes reported by included studies? (Including BOTH PA outcome and measurement instrument)	Yes / No / Unclear

7	Was the scientific quality of the included studies assessed and documented?	A priori' methods of assessment should be provided (e.g., for effectiveness studies if the author(s) chose to include only randomized, double-blind, placebo controlled studies, or allocation concealment as inclusion criteria); for other types of studies alternative items will be relevant.	1) Was the scientific quality of included studies assessed? (with a tool appropriate for the study type)<br>2) Was this done by at least 2 independent reviewers?<br>3) Was the scientific quality of studies documented?	Yes / No / Unclear
8	Was the scientific quality of the included studies used appropriately in formulating conclusions?	The results of the methodological rigor and scientific quality should be considered in the analysis and the conclusions of the review, and explicitly stated in formulating recommendations.	The results of the methodological rigor and scientific quality should be considered in the discussion of the results/conclusions of the review, and/or explicitly stated in formulating recommendations.	Yes / No / Not applicable / Unclear
9a	Were the methods used to combine the findings of studies appropriate?	For the pooled results, a test should be done to ensure the studies were combinable, to assess their homogeneity (i.e., Chi-squared test for homogeneity). If heterogeneity exists a random effects model should be used and/or the clinical appropriateness of combining should be taken into consideration (i.e., is it sensible to combine?).	1) Were the methods used to combine the findings of studies clearly described and/or referenced to appropriate text?	Narrative only / Correlate table with summary codes / Correlate table with summary codes and p-values by study type / Meta-analysis / Compiled by study type and outcome / List of supporting studies

9b			2) Is a test of heterogeneity reported? (to determine whether or not to do a meta-analysis) OR was heterogeneity discussed in reference to combining results?<br><br>3) Have the authors stated a definition of statistical heterogeneity?<br><br>4) If heterogeneity is present or suspected, has a random-effects model been used?<br><br>If not a meta-analysis, only 2 is sufficient for a "yes"; If a meta-analysis, all are required for a "yes"	Yes / No / Not applicable / Unclear
10	Was the likelihood of publication bias assessed?	An assessment of publication bias should include a combination of graphical aids (e.g., funnel plot, other available tests) and/or statistical tests (e.g., Egger regression test).	10.1 Was the likelihood of publication bias assessed (or considered in forming conclusions or recommendations)?	Yes, with a statistical test / Yes / No Unclear
11	Was the conflict of interest stated?	Potential sources of support should be clearly acknowledged in both the systematic review and the included studies.	Was there a conflict of interest statement?	Yes / No / Unclear

References

1. Pollock, A., et al., *Interventions for improving upper limb function after stroke.* Cochrane Database of Systematic Reviews, 2014(11).
2. Shea, B.J., et al., *Development of AMSTAR: a measurement tool to assess the methodological quality of systematic reviews.* BMC medical research methodology, 2007. **7**(1): p. 1-7.
